# Effects of Stem Density on Crown Architecture of Scots Pine Trees

**DOI:** 10.3389/fpls.2022.817792

**Published:** 2022-03-09

**Authors:** Ninni Saarinen, Ville Kankare, Saija Huuskonen, Jari Hynynen, Simone Bianchi, Tuomas Yrttimaa, Ville Luoma, Samuli Junttila, Markus Holopainen, Juha Hyyppä, Mikko Vastaranta

**Affiliations:** ^1^School of Forest Sciences, University of Eastern Finland, Joensuu, Finland; ^2^Natural Resources Institute Finland, Helsinki, Finland; ^3^Department of Forest Sciences, University of Helsinki, Helsinki, Finland; ^4^Department of Remote Sensing and Photogrammetry, Finnish Geospatial Research Institute, Masala, Finland

**Keywords:** growth and yield, terrestrial laser scanning, ground-based LiDAR, pipe-model theory, silviculture, forest management, thinning

## Abstract

Trees adapt to their growing conditions by regulating the sizes of their parts and their relationships. For example, removal or death of adjacent trees increases the growing space and the amount of light received by the remaining trees enabling their crowns to expand. Knowledge about the effects of silvicultural practices on crown size and shape and also about the quality of branches affecting the shape of a crown is, however, still limited. Thus, the aim was to study the crown structure of individual Scots pine trees in forest stands with varying stem densities due to past forest management practices. Furthermore, we wanted to understand how crown and stem attributes and also tree growth affect stem area at the height of maximum crown diameter (SAHMC), which could be used as a proxy for tree growth potential. We used terrestrial laser scanning (TLS) to generate attributes characterizing crown size and shape. The results showed that increasing stem density decreased Scots pine crown size. TLS provided more detailed attributes for crown characterization compared with traditional field measurements. Furthermore, decreasing stem density increased SAHMC, and strong relationships (Spearman’s correlations > 0.5) were found between SAHMC and crown and stem size and also stem growth. Thus, this study provided quantitative and more comprehensive characterization of Scots pine crowns and their growth potential. The combination of a traditional growth and yield study design and 3D characterization of crown architecture and growth potential can open up new research possibilities.

## Introduction

Trees are direct available resources to reproduction and growth and can regulate their size and the relationship between their parts. That way, trees adapt to changes in their growing conditions. The size of a tree correlates with the space a tree occupies and it defines tree growth that is linked to carbon sequestration (Vanninen and Mäkelä, 2000, 2005; Rayment et al., 2002; Pretzsch et al., 2015). Removal or death of trees enhances the light regime and photosynthesis for the remaining trees, which increases the crown size. This is particularly evident near the lowest limit of live crown where changes in the amount of light increase considerably more compared to the top of a tree (Oker-Blom and Kellomäki, 1982; Messier and Nikinmaa, 2000; Ilomaki et al., 2003; Niinemets, 2010).

Trees of different species require differing amount of growing space; birch (*Betula* sp.) requires more space than Scots pine (*Pinus sylvetris* L.), which in turn is more demanding than Norway spruce [*Picea abies* (H. Karst) L.] (Aaltonen, 1925; Pretzsch et al., 2015). Tolerant species [e.g., sugi (*Cryptomeria japonica* D. Don], eastern white pine [*Pinus strobus* L.)], response to light condition and modify their crown architecture (Hashimoto, 1990; O’Connell and Kelty, 1994). Mitchell (1969) reported that growth of branches is similar on the free side of a white spruce [*Picea glauca* (Moech) Voss] to a completely free-growing white spruce. Additionally, crown architecture (e.g., crown width and live-crown length) varies between mixed stands compared to monocultures (Bauhus et al., 2004; Bayer et al., 2013; Dieler and Pretzsch, 2013; Pretzsch, 2014). There is a relationship between tree size and growing conditions that can be assessed through the light regime. In dense forests, lower branches die due to the limited amount of light (Heikinheimo, 1953; Flower-Ellis et al., 1976; Kellomäki, 1980) specifically for light-demanding species such as Scots pines and birches (Kellomäki and Tuimala, 1981) and also loblolly pine (*Pinus taeda* L.) (Zeide, 1998), and this decreases live-crown ratio (i.e., proportion of live crown from tree height). Raulier et al. (1996) discovered that stand structure did not affect crown adjustment of black spruce [*Picea mariana* (Mill.) BSP].

Forest management is mainly aimed at increasing size and quality of the trees left to grow by regulating stand density and thus improving their growing conditions. First commercial thinning is especially important for Scots pines, and later thinnings, even if intensive, do not offer recovery from reduced live-crown ratio as it has been shown to reduce up to 37% of tree height (Mäkinen and Isomäki, 2004). The crowns of young trees recover better compared to old trees because height growth of young trees increases the length of live crown (Hynynen, 1995). In mature and old trees, height growth is slower, and recovery of a crown is limited to increasing the width and the number of leaves or needles. However, knowledge about the effects of silvicultural practices on more sophisticated crown attributes such as volume and also crown diameter and its variation that affects the shape of a crown is still limited. In addition, crown attributes from standing trees have mainly been limited to crown-base height, crown length, and live-crown ratio as adequate measurement techniques have been lacking.

Laser scanning (or light detecting and ranging LiDAR) has provided new opportunities for characterizing trees in more detail in three-dimensional space. Specifically, terrestrial laser scanning (TLS) has increasingly been used in producing a variety of tree attributes (Seidel et al., 2011, 2015; Metz et al., 2013; Saarinen et al., 2017; Hess et al., 2018; Chianucci et al., 2020; Georgi et al., 2021; Owen et al., 2021; Rais et al., 2021; Zhu et al., 2021). One of the challenging stem-related attributes to be measured from standing trees has been taper curve (i.e., diameters at various heights of a stem), and TLS data have been shown to overcome that challenge (Liang et al., 2014; Yrttimaa et al., 2019, 2020). Additionally, versatile crown attributes such as volume (Fernández-Sarría et al., 2013), surface area (Metz et al., 2013), asymmetry (Seidel et al., 2011), and height of the maximum crown projection area (Seidel et al., 2011) have been generated. Binkley et al. (2013) and Forrester (2014) have stated that crown projection area and crown volume, which can be obtained with TLS data, can be used as proxies for leaf area and leaf biomass. Furthermore, crown surface area has been used as a proxy for the photosynthetically active surface of the tree (Seidel et al., 2019a). TLS has also been used for studying competition between species (Martin-Ducup et al., 2016; Barbeito et al., 2017; Juchheim et al., 2019; Pretzsch, 2019; Hildebrand et al., 2021), the effects of management intensity on tree structure (Juchheim et al., 2017; Georgi et al., 2018; Bogdanovich et al., 2021), and also structural complexity of individual trees (Seidel, 2018; Seidel et al., 2019b; Saarinen et al., 2021). Thus, TLS provides a vast range of opportunities for understanding tree growth.

There is a long history of research where the relationship between crown and stem dimensions has been investigated (Krajicek et al., 1961; Larson, 1963; Grinrich, 1967; Curtin, 1970; Seymour and Smith, 1987; Pamerleau-Couture et al., 2015; Montoro Girona et al., 2016, 2017). Process-based models simulate tree growth as a function of leaf biomass, in other words of their photosynthetic elements (e.g., Valentine and Mäkelä, 2005). Shinozaki et al. (1964), on the other hand, proposed a conceptual framework for the relationship between the amount of stem tissue and corresponding supported leaves known as the pipe-model theory (PMT). The idea behind the PMT is that certain amount of leaves needs mechanical support and also enough water and nutrient supply to be sustained. One of the PMT’s properties is a proportional relationship between conductive area of the stem at a certain height and the mass of foliage above. This is related to Pressler’s law in which a cross-sectional area of an annual increment of a stem-ring is proportional to the quantity of foliage above that point (Pressler 186, cited in Larson, 1963). The PMT is part of a branch of science called allometry that has widely used for exploring plant growth and development (Niklas, 1994; Le Roux et al., 2001; Niklas and Enquist, 2002; Fourcaud et al., 2008; McDowell and Allen, 2015). The PMT has inspired investigations to understand relationships that are related to the amount of foliage. It has been shown that the total cross-sectional area of living branches is strongly correlated with foliage mass (Vanninen et al., 1996; Ilomaki et al., 2003; Kantola and Mäkelä, 2004). Longuetaud et al. (2006) reported that statistically significant indicators for tree vitality were the total cross-sectional area of branches, height-diameter at breast height (DBH) ratio (i.e., height/DBH), and the relative and absolute height of the crown base. More specifically, Lehtonen et al. (2020) and Hu et al. (2020) found leaf biomass of Scots pine to be proportional to the stem cross-sectional area at the crown base. However, in both cases, the relationship was influenced by other factors, such as age, site type, and temperature. There are indeed criticisms on the validity of the PMT, for which we direct the reader to the extensive review from Lehnebach et al. (2018). In any case, if traditional empirical models are using DBH as a proxy for growth potential, the question still remains if diameter at the crown base (DCB) could be a more accurate predictor.

The aim of this study is to investigate how crown structure of individual Scots pine trees varies when growing in different conditions due to the intensity and type of past thinning treatments. It is hypothesized that crown size decreases with increasing stem density (H1) and increases when suppressed and codominant trees were removed (H2) due to decreasing and increasing growing space, respectively. Related to the PMT, the objective is to understand the relationship between stem area at the height of the maximum crown diameter (SAHMC) and crown and stem dimensions and also growth of the tree. This relates to the question of the usefulness of DCB as a proxy for growth potential as it is of renewed importance since new technology such as TLS can now estimate this parameter more easily.

## Materials and Methods

### Study Area

**FIGURE 1:**
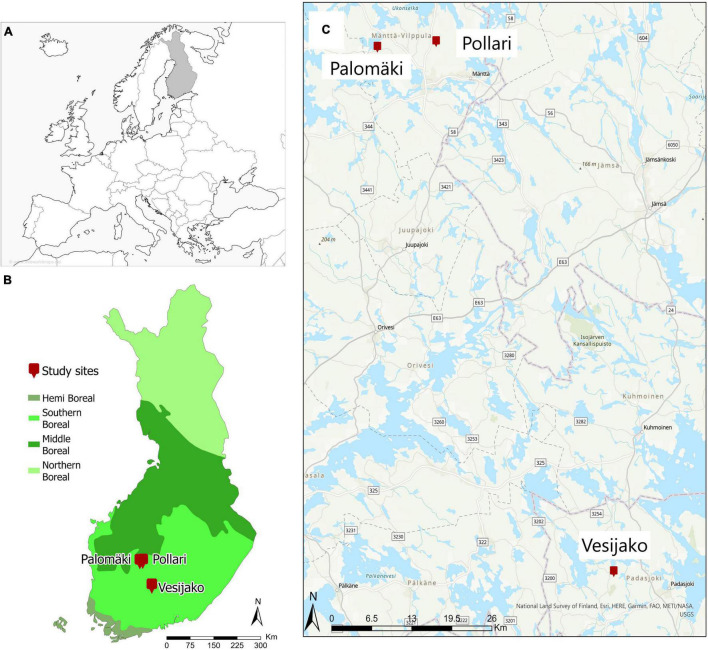
Location of tree study sites (i.e., Palomäki, Pollari, and Vesijako) and vegetation zones in Finland **(A,B)** and study sites on top of a base map **(C)**. © National Land Survey of Finland, Esri, HERE, Garmin, FAQ, METI/NASA, and USGS (www.freeworldmaps.net).

The study area is located in southern boreal forest zone in Finland and consists of three study sites (Palomkäi, Pollari, and Vesijako) (Figure 1) with relatively flat terrain (elevation above sea level ∼137 ± 17 m) in mesic heath forest [i.e., Myrtillus forest site type according to Cajander (1909)] dominated by Scots pine. The study sites were established and are maintained by the Natural Resources Institute Finland (Luke). The Palomäki study site was established in 2005, whereas Pollari and Vesijako study sites were established in 2006. The temperature sum for Palomäki, Pollari, and Vesijako are 1,195, 1,130, and 1,256 degree days, respectively. At the time of the establishment, the stand age was 50, 45, and 59 years for Palomäki, Pollari, and Vesijako, respectively. The proportion of Norway spruce and deciduous trees (i.e., *Betula* sp and *Alnus* sp) from the total stem volume was 3.06 and 0.03%, respectively, in 2019.

### Sampling Protocol and Silvicultural Treatments

**FIGURE 2:**
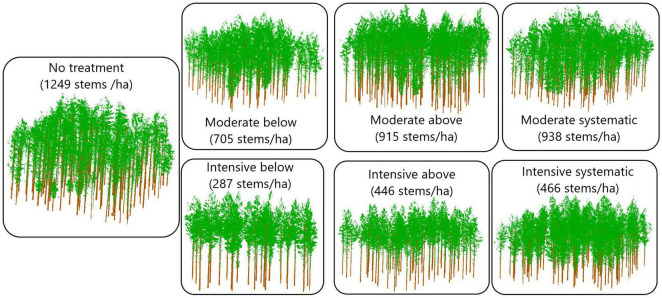
Schematic representation of the effects of silviculture treatments on stand density based on TLS data collected in October 2018.

Nine rectangular sample plots (sized 1,000–1,200 m^2^) were placed on each study site, and first in situ measurements were carried out at the same time. The experimental study design included two levels of thinning intensity and three thinning types (Figure 2), which resulted in six different thinning treatments (i.e., moderate from below, moderate from above, moderate systematic, intensive from below, intensive from above, and intensive systematic) that were replicated from one to two times in each study site using a randomized block design. One plot at each study site was left as a control plot where no thinning has been carried out since the establishment of the sites. Finally, there were four plots with either moderate or intensive thinning from below, five plots with either moderate or intensive systematic thinning, three plots with moderate or intensive thinning from below and also three control plots.

Thinning intensity was defined as the remaining basal area whereas thinning type determined which trees (based on a crown class) were removed. The remaining relative stand basal area after moderate thinning was ∼68% of the stocking before thinning and intensive thinning reduced the stocking levels down to 34%. Suppressed and codominant trees were removed in thinning from below whereas dominant trees were mainly removed in thinning from above. Dominant trees were removed and small, suppressed trees were left to grow in systematic thinning without considering regular spatial distribution of the remaining trees, which was considered in thinnings from below and above. Additionally, unsound and damaged trees (e.g., crooked and forked) were removed in thinnings from below and above.

Tree species, DBH from two perpendicular directions, crown layer, and health status were recorded for each tree within a plot during all in situ measurements (i.e., at the establishment, 10 years after the establishment, and between October 2018 and April 2019 for this study). Each sample plot also includes ∼22 sample trees from which also tree height, live-crown-base height, and height of the lowest dead branch were measured. Plot-level attributes before and after thinning treatments (i.e., at the establishment) and also based on the in situ measurements in 2018–2019 are presented in Table 1, and the development of tree-level attributes for each thinning treatment can be found in Table 2.

**TABLE 1:** Mean and standard deviation (with ±) of stand characteristics by treatments before and after the thinning treatments (2005–2006) and also after the growth period (2018–2019).

	No treatment	Moderate below	Moderate above	Moderate systematic	Intensive below	Intensive above	Intensive systematic
**Before thinning (2005–2006)**							
G (m^2^/ha)	27.6 ± 6.7	26.9 ± 6.9	27.8 ± 2.0	25.4 ± 2.6	26.9 ± 4.5	24.7 ± 2.6	20.6 ± 4.2
N/ha	1336 ± 97	1285 ± 270	1417 ± 419	1256 ± 129	1260 ± 158	1201 ± 78	1218 ± 333
V (m^3^/ha)	224.0 ± 93.4	215.4 ± 70.4	216.9 ± 18.7	199.7 ± 42.4	216.6 ± 55.4	191.0 ± 35.6	210.6 ± 62.3
D_*w*_ (cm)	17.8 ± 3.4	17.5 ± 2.1	17.3 ± 1.7	17.5 ± 1.6	18.0 ± 2.1	17.6 ± 1.3	18.0 ± 3.6
H_*w*_ (m)	16.1 ± 3.3	16.1 ± 1.8	15.9 ± 1.1	15.9 ± 2.1	16.3 ± 1.8	15.6 ± 1.6	16.2 ± 2.9
**After thinning (2005–2006)**							
G (m^2^/ha)	27.6 ± 6.7	18.3 ± 2.1	18.5 ± 1.1	18.2 ± 1.1	8.9 ± 0.8	9.1 ± 0.8	8.7 ± 0.7
N/ha	1336 ± 97	719 ± 130	955 ± 258	988 ± 129	292 ± 55	479 ± 113	522 ± 183
V (m^3^/ha)	224.0 ± 92.8	148.8 ± 30.2	144.0 ± 15.3	141.3 ± 23.6	72.9 ± 12.4	69.1 ± 11.3	67.3 ± 14.7
D_*w*_ (cm)	17.8 ± 3.4	18.7 ± 2.4	16.9 ± 1.9	16.5 ± 1.6	20.4 ± 2.7	16.5 ± 2.5	15.7 ± 3.0
H_*w*_ (m)	16.1 ± 3.3	16.5 ± 1.9	15.7 ± 1.2	15.6 ± 2.1	16.9 ± 1.8	15.3 ± 1.9	15.5 ± 2.7
**After growth period (2018–2019)**							
G (m^2^/ha)	37.1 ± 4.6	28.4 ± 2.5	28.3 ± 28.3	27.6 ± 1.8	15.9 ± 0.7	16.1 ± 1.2	15.9 ± 1.6
N/ha	1249 ± 159	705 ± 113	915 ± 214	938 ± 111	287 ± 65	446 ± 82	466 ± 172
V (m^3^/ha)	380.3 ± 93.9	291.8 ± 44.7	282.3 ± 6.1	267.9 ± 16.1	160.8 ± 9.1	150.5 ± 12.6	150.4 ± 9.9
D_*w*_ (cm)	21.2 ± 3.0	23.5 ± 2.2	21.2 ± 1.9	20.7 ± 1.2	27.5 ± 3.1	22.3 ± 2.1	22.2 ± 3.0
H_*w*_ (m)	21.3 ± 3.1	21.7 ± 2.0	21.0 ± 1.1	20.3 ± 1.4	21.6 ± 1.6	19.5 ± 1.2	20.0 ± 2.2

*G, basal area; N, stem number per hectare; V, volume; D_w_, mean diameter weighted by basal area; H_w_, mean height weighted by basal area.*

**TABLE 2:** Mean tree-level attributes with their standard deviation (with ±) for each treatment at the year of the establishment (2005–2006) and after the growth period (2018–2019).

	No treatment	Moderate below	Moderate above	Moderate systematic	Intensive below	Intensive above	Intensive systematic
**2005–2006**							
DBH (cm)	15.4 ± 4.6	17.6 ± 3.3	15.3 ± 3.3	14.8 ± 3.5	19.3 ± 3.4	15.1 ± 3.1	14.8 ± 4.1
Height (m)	14.7 ± 2.6	15.9 ± 1.9	15.3 ± 1.2	14.6 ± 1.9	16.5 ± 1.8	14.8 ± 1.8	14.7 ± 2.6
Volume (dm^3^)	160.5 ± 119.7	202.7 ± 89.3	149.6 ± 76.2	138.1 ± 77.8	249.2 ± 107.0	141.8 ± 73.4	145.6 ± 97.1
**2018–2019**							
DBH (cm)	18.7 ± 5.0	22.2 ± 3.7	19.3 ± 4.3	18.8 ± 4.2	26.4 ± 3.9	21.1 ± 3.5	20.8 ± 4.3
Height (m)	20.2 ± 3.0	21.2 ± 2.1	20.4 ± 1.6	19.4 ± 2.2	21.2 ± 1.7	19.1 ± 1.5	19.6 ± 2.8
Volume (dm^3^)	299.4 ± 190.8	408.3 ± 106.3	306.4 ± 145.6	282.5 ± 137.2	563.8 ± 202.5	335.2 ± 125.4	347.0 ± 173.3

*DBH, diameter at breast height.*

### Terrestrial Laser Scanning Data

**FIGURE 3:**
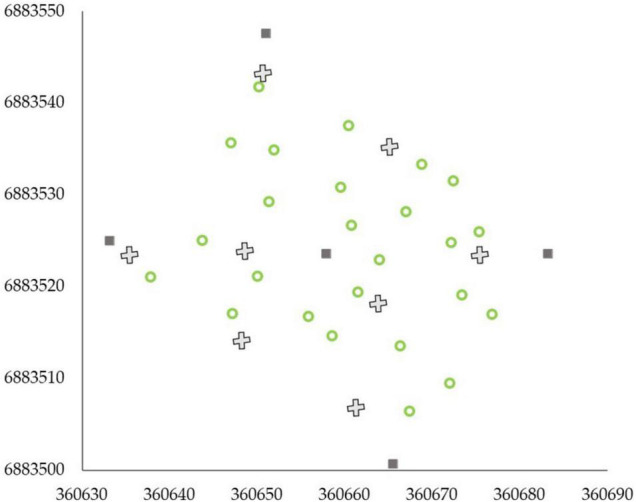
Scan design of eight scans (denoted as X) per an example sample plot. Dark gray squares represent the plot corners and center and green circles trees within the plot. *X* and *Y* axes present the coordinates of the sample plot in ETRS-TM35FIN projected coordinate system.

Terrestrial laser scanning data acquisition was carried out with a Trimble TX5 3D phase-shift laser scanner (Trimble Navigation Limited, United States) operating at a 1,550 nm wavelength and measuring 9,76,000 points per second. This resulted in a hemispherical (300° vertical × 360° horizontal) point cloud with a point distance approximately 6.3 mm at a 10-m distance. Eight scans were acquired from each sample plot between September and October 2018. Two scans were placed on two sides of the plot center, and six auxiliary scans were placed closer to the plot borders (Figure 3). Artificial targets (i.e., white spheres with a diameter of 198 mm) were placed around each sample plot to be used as reference objects for registering the eight scans into a single, aligned coordinate system with a FARO Scene software (version 2018). The automatic registration utilizing the reference targets resulted in a mean distance error of 2.9 ± 1.2 mm, with mean horizontal and vertical error of 1.3 ± 0.4 mm and 2.3 ± 1.2 mm, respectively, provided by the FARO Scene software for each reference target. LAStools software (Isenburg 2019) was used to normalize the point heights to heights above ground by applying a point cloud normalization workflow presented by Ritter et al. (2017).

First, plot-level TLS point clouds were segmented to identify points from individual trees. Local maxima from canopy height models (CHMs) with a 20-cm resolution were identified using the Variable Window Filter approach (Popescu and Wynne, 2004), and the Marker-Controlled Watershed Segmentation (Meyer and Beucher, 1990) was applied to delineate crown segments. A point-in-polygon approach was applied for identifying all points belonging to each crown segment. To identify points that originated from stem and crown within each crown segment, a point cloud classification procedure by Yrttimaa (2021) was used. The classification of stem and non-stem points assumed that stem points have more planar, vertical, and cylindrical characteristics compared to non-stem points that represent branches and foliage (Liang et al., 2014; Yrttimaa et al., 2020). The method by Yrttimaa et al. (2019, 2020) is an iterative procedure beginning from the base of a tree and proceeding toward treetop. More detailed description of the point cloud classification workflow can be found in Yrttimaa et al. (2019, 2020). The result of this step was 3D point clouds for each individual Scots pine tree (*n* = 2,174) within the 27 sample plots.

### Data Analyses

We investigated several traits characterizing crown size and shape (Table 3). Points from TLS that were classified originating from branches and foliage (i.e., crown points) in the previous step were utilized. A 2D convex hull was fitted to envelope the crown points of each tree of which crown projection area was derived. Crown diameter, on the other hand, was defined as the distance between the two most outer points in xy-space of the 2D convex hull. To obtain crown volume and surface area, a 3D convex hull was fitted to the crown points. We also wanted to investigate crown shape and thus divided the crown points into height percentiles (i.e., slices) of 10% starting from the lowest points. Then, 2D convex hull was fitted for each slice and its area and diameter were similarly obtained to the maximum crown diameter. Furthermore, mean, standard deviation, and range (i.e., crown taper) of these slice diameters were saved.

**TABLE 3:** Crown traits and how they were defined and/or generated from TLS point clouds.

Trait	Definition/Calculation
Projection area	Area of the maximum crown diameter from 2D convex hull
Crown volume	Calculated using 3D convex hull
Surface area	Surface area of the 3D convex hull
Crown diameters	Crown points were divided in height percentiles (i.e., slices) of 10% starting from the lowest part and their diameter was calculated using 2D convex hull
Maximum crown diameter	Maximum diameter based on the 2D convex hull of the crown slices
Mean crown diameter	Mean diameter of the crown slices
Standard deviation of crown diameter	Standard deviation of the diameters of the crown slices
Height at the maximum crown diameter (HMC)	Defined from the crown slices
Crown length	Distance between the HMC and tree height
Crown tapering	Difference between maximum and minimum diameter of the crown slices
Live-crown ratio	Proportion of crown length from the tree height
Stem area at the height of the maximum crown diameter (SAHMC)	Stem diameter at the HMC was obtained from the taper curve and basal area was then calculated as pi/4*d^2^

Height of the maximum crown diameter (HMC) from TLS was used to define crown length (i.e., live-crown-base height was deducted from tree height) and live-crown ratio (i.e., proportion of crown length from tree height). Finally, stem diameter at the HMC was obtained from the taper curve, and stem area at the SAHMC was calculated as pi/4*d^2^.

Traits characterizing stem included DBH, stem volume, height-DBH ratio (i.e., height/DBH), and cumulative volume. Tree height was obtained using the height of the highest TLS point of each tree (i.e., normalized above ground) whereas DBH was defined from taper curve obtained with a combination of circle fitting to original stem points and fitting a cubic spline (see Yrttimaa et al., 2019; Saarinen et al., 2020). Stem volume, on the other hand, was defined by considering the stem as a sequence 10-cm vertical cylinders and summing up the volumes of the cylinders using the estimated taper curve. Finally, cumulative stem volume was calculated as the height at which 50% of stem volume was accumulated.

As TLS data were only available for one time point, in situ measurements were utilized for obtaining growth information of individual Scots pine trees. Growth of DBH, tree height, stem volume, and change in height/DBH were calculated using in situ measurements conducted in 2005–2006 (i.e., at the time of establishment of the study sites) and 2018–2019 (i.e., the latest in-situ measurements) for all live Scots pine trees that were identified from the sample plots during the latest field measurements.

Due to the data structure (i.e., several sample plots in each study site), a nested two-level linear mixed-effects model (Equation 1) was fitted using restricted maximum likelihood included in package “nlme” (Pinheiro et al., 2013) of the R software to assess the effects of thinning treatment on crown, stem, and growth traits and also on SAHMC.


(1) $$y_{\mathrm{ij}}=\beta_{1}\,\mathrm{Moderate}\,{\mathrm{below}}_{i}+\beta_{2}\,\mathrm{Moderate}\,{\mathrm{above}}_{i}+\beta_{3}\,\mathrm{Moderate}\,{\mathrm{systematic}}_{i}+\beta_{4}\,\mathrm{Intensive}\,{\mathrm{below}}_{i}+\beta_{5}\,\mathrm{Intensive}\,{\mathrm{above}}_{i}+\beta_{6}\,\mathrm{Intensive}\,{\mathrm{systematic}}_{i}+\beta_{7}\,N\,o\,t\,r\,e\,a\,t\,m\,e\,n\,t_{i}+a_{j}+c_{i\,j}+\epsilon_{i\,j},$$

where, *y*_*ij*_ is each crown, stem, and growth trait and also SAHMC at a time, β_1_,…β_7_ are fixed parameters, i = 1, …, M, refers to study site, j = 1, …, *n_i_*, to a plot, *a_j_* and *c*_*ij*_ are normally distributed random effects for sample plot *j* and for sample plot *j* within study site *i*, respectively, with mean zero and unknown, unrestricted variance–covariance matrix, and ϵ*ij* is a residual error with a mean zero and unknown variance. The random effects are independent across study sites and sample plots and also residual errors are independent across trees. The effects of a study site and a sample plot within the study sites on crown, stem, and growth traits and also on SAHMC were assessed through their variances. Furthermore, we used Tukey’s honest significance test to reveal possible statistically significant differences in crown, stem, and growth traits and also in SAHMC between different thinning treatments.

Correlations between dependent and independent variables were investigated using Pearson’s correlation coefficient. Furthermore, the significance level of the correlation was investigated. The nested two-level linear mixed-effect model in Equation 1 was utilized in investigating the possible relationship between SAHMC and different crown, stem, and growth traits. Each crown (Table 2), stem (i.e., DBH, stem volume, and height/DBH), and growth (ΔDBH, Δtree height, Δstem volume, and Δheight/DBH) trait was separately used in Equation 1 as a single predictor variable.

## Results

### The Effects of Stem Density on Crown Architecture

**FIGURE 4:**
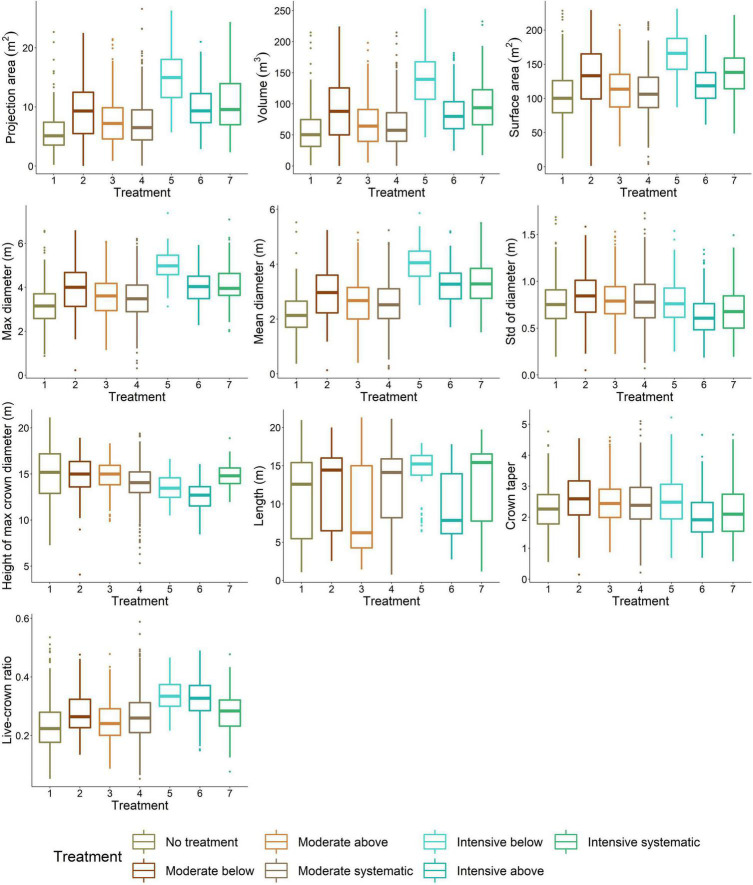
Variation in crown attributes between thinning treatments. 1, no treatment (i.e., control); 2, moderate thinning from below; 3, moderate thinning from above; 4, moderate systematic thinning from above; 5, intensive thinning from below; 6, intensive thinning from above; and 7, intensive systematic thinning from above.

**FIGURE 5:**
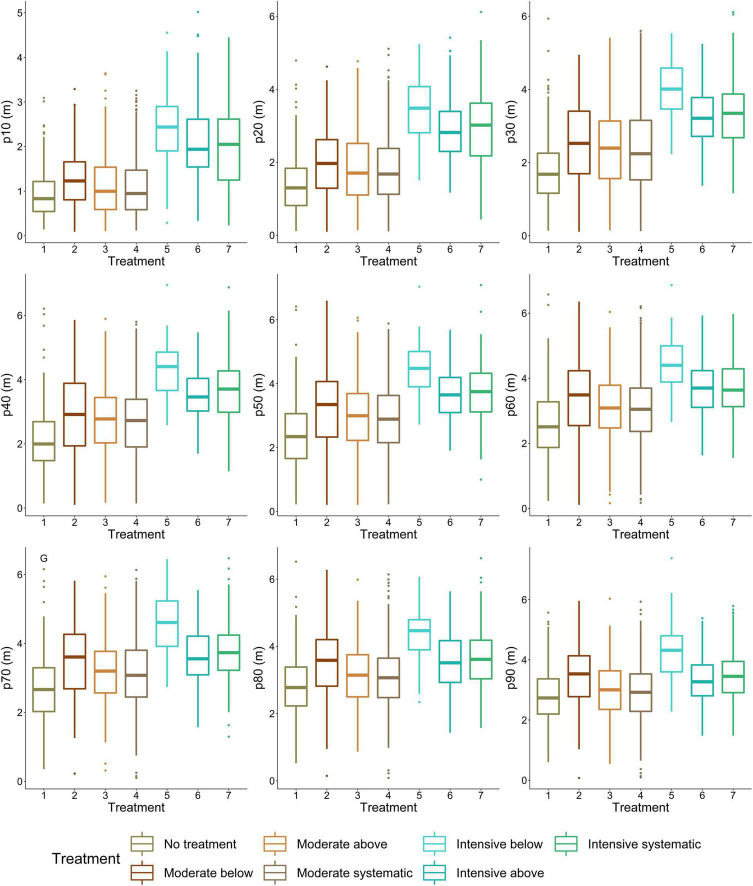
Variation in crown diameter at height percentiles between thinning treatments. P10 indicates the lowest height percentile (i.e., the most bottom part of a crown), whereas p100 is the highest height percentile (i.e., the highest part of a crown). 1, no treatment (i.e., control); 2, moderate thinning from below; 3, moderate thinning from above; 4, moderate systematic thinning from above; 5, intensive thinning from below; 6, Intensive thinning from above; and 7, intensive systematic thinning from above.

Difference in stem density/ha varied from 430 to 470 between moderate and intensive thinning and from 310 to 960 stem/ha between no treatment and thinned (i.e., all other) plots. When thinning intensity increased (i.e., stem density/ha decreased) from moderate to intensive thinning from below, crown volume, projection area, and maximum and mean diameter increased (Figure 4) statistically significantly (*p* < 0.05). Similarly, live-crown ratio and also crown diameter at the bottom of a crown (i.e., 10–30 percentiles) (Figure 5) statistically significantly (*p* < 0.05) increased when thinning intensity increased, but this was true for all thinning types. However, there was no statistically significant (*p* > 0.05) difference in crown traits between of moderate thinnings and no treatment.

**FIGURE 6:**
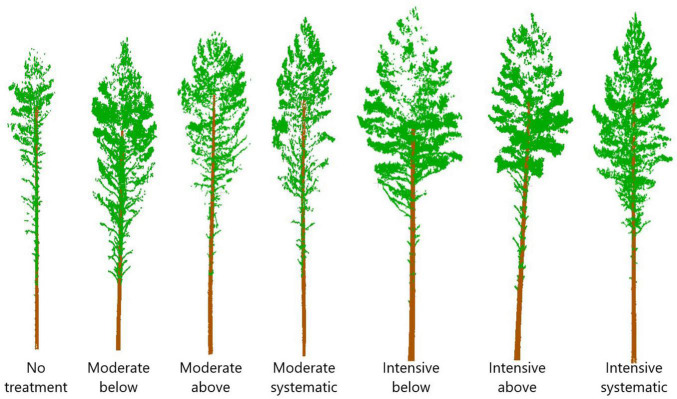
Point clouds from example trees from different thinning treatments. Stem densities of the treatments were on average ∼1,250, 720, 910, 940, 290, 450, and 470 stems/ha for no treatment, moderate below, moderate above, moderate systematic, intensive below, intensive above, and intensive systematic, respectively.

Thinning type (i.e., removal of suppressed and codominant or dominant trees) had a less clear effect on crown size and shape. Statistically significant (*p* < 0.05) differences were only present in crown volume, surface and projection area, maximum and mean diameter, and also diameters at the top part of a crown when intensive thinning from below was compared with other intensive thinnings (difference in stem density/ha between 20 and 180). In other words, in intensive thinning crown attributes were significantly larger when suppressed and codominant trees had been removed (i.e., thinning from below) compared to when dominant trees were removed (i.e., thinning from above and systematic thinning). This is also visible for example trees from different thinning treatments (Figure 6).

### The Effects of Stem Density on Stem Area at the Height of Maximum Crown Diameter

**FIGURE 7:**
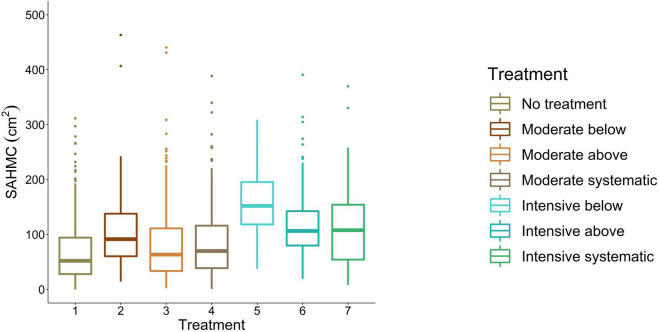
SAHMC between thinning treatments. 1, no treatment (i.e., control); 2, moderate thinning from below; 3, moderate thinning from above; 4, moderate systematic thinning from above; 5, intensive thinning from below; 6, intensive thinning from above; and 7, intensive systematic thinning from above.

Stem area at the height of maximum crown diameter ranged from 67.4 cm^2^ to 170.2 cm^2^ being the smallest with no treatment and the largest with intensive thinning from below (Figure 7). For moderate thinnings, SAHMC was 90.6 cm^2^, on average, whereas with intensive thinnings, it was 132.2 cm^2^. Lower stem densities increased SAHMC, and SAHMC was statistically significantly (*p* < 0.05) greater when stem density increased from ∼290 stems/ha (i.e., intensive below) to at least ∼720 stems/ha (i.e., moderate below). In other words, SAHMC was statistically significantly different between intensive thinning from below and all other thinning treatments, which include no treatment, except between intensive thinning from above.

### Relationship Between Stem Area at the Height of Maximum Crown Diameter and Crow and Stem Attributes as Well as Tree Growth

There was high correlation (≥0.5|) between SAHMC and crown volume (Table 4). Specifically, traits characterizing stem size (i.e., DBH, stem volume, and height at which 50% of stem volume accumulated) and size growth (i.e., DBH growth and stem volume growth) showed high positive correlation (>0.5). Height/DBH ratio, on the other hand, showed negative correlation with SAHMC. Correlations between SAHMC and all crown, stem, and growth attributes were statistically significant.

**TABLE 4:** Pearson’s correlation coefficients between SAHMC diameter and crown, stem, and growth attributes and also coefficient value from the nested two-level linear mixed-effect models where each trait was independently included as a single predictor variable against SAHMC.

	Trait	Pearson’s correlation coefficient	Coefficient value
Crown attributes	Projection area	0.49*	6.32*
	Crown volume	0.50*	0.72*
	Surface area	0.47*	0.78*
	Maximum crown diameter	0.45*	24.61*
	Mean crown diameter	0.47*	28.68*
	Standard deviation of crown diameter	0.09*	29.89*
	Height at the maximum crown diameter	−0.36*	−14.34*
	Crown length	0.20*	13.52*
	Crown tapering	0.14*	12.46*
	Live-crown ratio	0.66*	521.22*
Stem attributes	DBH	0.66*	10.40*
	Stem volume	0.70*	0.32*
	Height/DBH	−0.61*	−167.17*
	Relative stem volume	0.38*	310.10*
	Height at which 50% of stem volume accumulated	0.70*	12.71*
Growth attributes	DBH growth	0.59*	21.85*
	Height growth	0.23*	21.24*
	Stem volume growth	0.67*	0.52*
	Change in height/DBH	0.35*	215.64*

DBH, diameter at breast height. * denotes statistically significant correlation or importance in the model.

*Correlation ≥ |0.5| are bolded.*

Crown diameters at different heights also showed positive correlation (≥0.4) with SAHMC. Furthermore, the results from the nested two-level linear mixed-effect model showed that increment in most of the crown, stem, and growth attribute, when independently included as a predictor variable, increased SAHMC. HMC and height/DBH were exceptions as their increment decreased SAHMC. Increasing live-crown ratio, relative stem volume, and change in height/DBH increased SAHMC ten times more than other crown, stem, and growth attributes, whereas the effect of increasing height/DBH was of similar magnitude but to different directions, in other words, it decreased SAHMC. When each trait characterizing crown, stem, and growth was separately added as a predictor variable to estimate SAHMC, each of them was statistically significant (*p* < 0.001) for the models (Table 4).

## Discussion

The results showed how thinning treatments carried out >10 years ago affected crown shape and size of Scots pine trees. As stem density decreased, crown volume, surface area, and maximum diameter increased. Also, diameter of the lower part of a crown (<80th height percentile) increased with decreasing stem density. These results suggest that stem density affects crown shape and size of Scots pine trees in boreal forests. Lower stem densities (i.e., ≤700 stems/ha) also increased SAHMC. Furthermore, when crown and stem size and also stem growth increased, SAHMC also grew.

One of the traditional parameters used for characterizing crown architecture is live-crown ratio, and the results here showed that it differed between stem densities, similar to the findings by Kellomäki and Tuimala (1981), Clark Baldwin et al. (2000), and Tahvanainen and Forss (2008). Mäkelä (1997) presented a model where lower stem densities led to larger live-crown ratio, whereas Vanninen (2004) developed a model for aboveground growth allocation and found that increasing crown ratio increased growth allocation to branches. Live-crown ratio has also been used as a measure for growth and tree vigor (Dyer and Burkhart, 1987; Zarnoch et al., 2004). Mäkelä and Vanninen (2001) reported that the maximum foliage density was lower in height for dominant Scots pine trees compared to suppressed, whereas in our study, lower part of a crown was similar in size for all social crown classes but upper part was significantly larger for Scots pines that were originally considered as dominant trees and were left to grow in the sparsest plots (i.e., intensive thinning carried out). We also found significant differences between advanced crown traits (namely crown surface area and volume) at least among the sparsest stem densities (i.e., intensive thinning). Finally, the study confirmed the results presented by Oker-Blom and Kellomäki (1982) as the lowest part of a Scots pine tree crown was larger in low stem densities. Our study enabled assessing crown architecture for 2,174 live Scots pine trees; thus, the use of TLS for obtaining enhanced information on canopy structure and architecture can be justified.

There is uncertainty in the SAHMC as the HMC may not represent the height of crown-base height, which is traditionally used for crown length and live-crown ratio. Thus, also SAHMC may not represent the true DCB. However, it has not been traditionally feasible to measure DCB from standing trees, whereas measurements on stem diameters from TLS data offer this. Thus, our results show a way toward assessing the usefulness of DCB as a proxy for growth potential of individual trees. There was strong correlation (≥0.5) between SAHMC and crown volume but also with DBH and stem volume, and their growth. This indicates that DCB or SAHMC could also be used when assessing growth potential, and TLS offers a means for obtaining this information.

Studies utilizing TLS in assessing tree development include European beech [*Fagus sylvatica* (L.)] (Juchheim et al., 2017; Georgi et al., 2018) and holm oak (*Quercus ilex* L.) (Bogdanovich et al., 2021). Juchheim et al. (2017) found that increasing thinning intensity increased crown surface area of European beech, which is in line with our results for Scots pine. Georgi et al. (2018) reported that crown size (i.e., crown volume, projection area, surface area, length, and live-crown ratio) of European beech trees growing in stands without forest management in ≥50 years was statistically significantly lower compared to European beech trees growing in managed stands or stands with ≤20 years without forest management. Our results showed that intensive thinning resulted in statistically significant difference in crown traits (e.g., crown volume, projection area, and maximum and mean diameter) when compared to moderate thinning and no treatment. However, moderate thinning had no effect on crown size when compared to no treatment. Our previous study utilizing the same dataset showed no statistically significant difference between traits characterizing stem size and shape between moderate thinning and no treatment (Saarinen et al., 2020). This, together with the results from this study, suggests that although moderate thinning increased the growing space of the remaining trees, the difference in stem density (i.e., ∼310–540 stems/ha) did not lead to a statistically significant growth response of individual Scots pine trees. When the difference in stem density was almost the double (i.e., intensive thinning), there was, however, a statistically significant difference int the growth response of Scots pine crowns.

As height/DBH and absolute height of the crown base have been identified as indicators for tree vitality (Longuetaud et al., 2006), this study presented a means for obtaining those attributes. Height/DBH has been shown to increase as forest management intensity increased (Saarinen et al., 2020), whereas HMC did not differ significantly between tree densities in this study. However, this study provided DCB and stem cross-sectional area at the HMC, which enables studies on their suitability as proxies for growth potential. Competition between trees can be regulated through forest management, and although competition was not studied here, stem density provides an indication for pressure trees encounter around them (Pretzsch, 2014). Competition in young stands is more intense compared to more old-growth stands (Brassard and Chen, 2006) where especially in natural stands dominant trees start to die (Chen and Popadiouk 2002). Trees with larger crowns have more foliage for photosynthesis, and they are thus larger in size (Zarnoch et al., 2004); results from this and our previous study (Saarinen et al., 2020) confirm this.

As TLS provides crown characteristics and also DCB and SAHMC, future studies could include them in growth models to understand their potential in predicting tree growth, and competition between trees could be studied through crown traits instead of stem dimensions (e.g., DBH), and are there differences in growth response of crown traits between tree species and geographical regions. This study only concentrated on Scots pine trees but the methodology can be applied to other tree species. This study was conducted in one study area, and the results may not apply to Scots pine trees growing in other vegetation zones or forest site types. Another limitation of this study is related to identify individual trees from TLS point clouds as there are uncertainties in the methodology, such as non-detection of trees and only a part of the points originating from a crown can correctly be identified (Yrttimaa et al., 2019, 2020). Nevertheless, as shown with other studies utilizing TLS data, it can be seen as a useful tool for providing crown traits from individual trees.

This study concentrated on investigating crown structure of individual Scots pine trees in different stem densities. Increasing stem density decreased crown size, confirming our hypothesis (H1). With low stem densities (i.e., intensive thinning), crown size also increased when suppressed and codominant trees were removed (i.e., thinning from below) partly confirming the H2 (i.e., no difference in moderate thinnings). Furthermore, a relationship between SAHMC and crown and stem attributes was found. Thus, this study showed how tree density affects crown shape and size of Scots pine trees and how they are adapted to the growing conditions of the trees. As stem density can be regulated through forest operations such as thinning, the results of this study can be utilized when planning management actions.

## Conclusion

Stem densities affected crown size and shape of Scots pine trees growing in boreal forests. When growing in a denser forest, the crown size of Scots pine tree decreased, which indicates more competition on light between adjacent tree crowns. Although this has been known for decades as growth and yield studies have a long history, this study provided quantitative attributes assessing crown size (e.g., crown volume, projection area, surface area, and diameter) and shape (i.e., diameters at different heights of a crown, their mean and standard deviation) of Scots pine trees. Additionally, the study provided stem diameter and cross-sectional area at the height of maximum crown diameter (i.e., SAHMC) that can be assumed to present crown-base height. Increasing forest management intensity increased the SAHMC, and there was strong relationship between it and crown, stem, and growth attributes. Thus, this study provided more insight on the effects of forest management on crown architecture of Scots pine trees, and it can be concluded that this study expanded our knowledge on the crown response of Scots pine trees to the past forest management activities. This was enabled with detailed 3D TLS data that offered quantitative and more comprehensive characterization of Scots pine crowns and growth potential. The novelty of the study is to couple a traditional growth and yield study design (i.e., two thinning intensities and three thinning types) with a 3D characterization of stem and crown of Scots pine trees with TLS. This type of combination can give answers to new questions related to forest and tree dynamics.

## Data Availability Statement

The raw data supporting the conclusions of this article are openly available at Zenodo (https://doi.org/10.5281/zenodo.5783404).

## Author Contributions

NS, VK, SH, JaH, and SB were involved in conceptualization. NS, TY, VK, and VL contributed in the data curation. NS performed formal analysis, investigated the study, contributed in project administration, visualized the study, and was involved in roles/writing – original draft. NS, SJ, MH, and JuH contributed in funding acquisition. NS and TY carried out methodology and validated the manuscript. SH, JaH, MH, and JuH provided the resources. TY provided the software. MV supervised the study. All authors participated in writing, reviewing, and editing.

## Conflict of Interest

The authors declare that the research was conducted in the absence of any commercial or financial relationships that could be construed as a potential conflict of interest.

## Publisher’s Note

All claims expressed in this article are solely those of the authors and do not necessarily represent those of their affiliated organizations, or those of the publisher, the editors and the reviewers. Any product that may be evaluated in this article, or claim that may be made by its manufacturer, is not guaranteed or endorsed by the publisher.
